# Public health facility vulnerabilities, preparedness, and health outcomes for *Plasmodium falciparum* and dengue virus-infected children under 5 years with acute febrile illnesses in Western Kenya

**DOI:** 10.3389/fpubh.2025.1526558

**Published:** 2025-07-23

**Authors:** Jack Ogony, Diana Menya, Judith Mangeni, George Ayodo, Simon Karanja

**Affiliations:** ^1^Department of Environmental Health and Disease Control, Jomo Kenyatta University of Agriculture and Technology, Nairobi, Kenya; ^2^Department of Epidemiology and Biostatistics, Moi University, Eldoret, Kenya; ^3^Department of Public Health and Community Development, Jaramogi Oginga Odinga University of Science and Technology, Kisumu, Kenya

**Keywords:** climate change, dengue virus, healthcare system, longer hospitalization, malaria, health outcome, preparedness, vulnerability

## Abstract

**Introduction:**

Climate change and infectious health risks are threatening healthcare systems, cascading into devastating consequences globally. This crisis is altering the footprints of many vector-borne disease control programs. Sub-Saharan countries face complex challenges as patterns of vector-borne diseases transform, causing more than 17% of the global mortality. Climate change-related disasters are increasing worldwide, with Sub-Saharan Africa being the most prone region. Although healthcare facilities should be on the front line in protecting lives, they are often under pressure and are vulnerable to extreme weather events. Public healthcare preparedness and the associated health outcomes are less frequently considered.

**Methodology:**

This was a three-month follow-up prospective cohort study that determined public health facility vulnerability, preparedness, and health outcomes through a questionnaire administered to facilities in charge, guardians of children seeking health services, and those with acute febrile illnesses. Key Informant Interviews were conducted with selected members of the County's Health Management Team.

**Results:**

A total of 378 participants were successfully followed. A total of 17 (81.0%) facilities were able to diagnose and treat malaria, while 4 (18.0%) were only able to diagnose and treat dengue virus cases. In Bunyala Sub-County, 6 of the 10 facilities were located on or near floodplains or wetlands, while 5 (45.0%) facilities in Kisumu had the same location. The longest hospitalizations (>5 days) were observed at the Kisumu site, while the highest recovery rate [184 (96.8%)] was noted in Bunyala Sub-County compared to 171 (91.0%) in Kisumu.

**Conclusion:**

Public health facilities are not only vulnerable but also unprepared to contain the rising climate change-driven infectious disease burden. Even though healthcare facilities are fairly able to diagnose and treat malaria, the majority lack the ability to diagnose and treat dengue fever. Longer hospitalization was highest among children diagnosed with dengue fever. There is a need for enhanced arboviral disease surveillance and policies on integrated multisectoral approaches to reduce health system vulnerabilities and increase preparedness.

## Introduction

The global impact of climate change on public health varies by region due to geographic differences and the capacity of countries to adapt to these impacts. More than three billion people live in areas that are highly vulnerable to climate change, thereby being exposed to higher health risks. The damage and impacts caused by extreme events in a changing climate are not only compromising the achievement of sustainable development goals but also wading off the hard-won development gains of the past (1). The climate crisis is pressing health systems in the Eastern and Southern Africa region (ESAR), which are already under significant pressure due to population growth, limited financial and human resource capacity, and further deterioration due to COVID-19's impacts (2). Sub-Saharan Africa is on the front line of the climate crisis, as the continent faces a plethora of interconnected threats, including climate extremes, epidemics, and environmental degradation, which have cascaded into devastating consequences, including loss of life and livelihoods, and displacement of large populations, among others (3). Threats come directly as extreme heat or indirectly, disrupting public and routine health services (4). Health emergencies affect millions of people annually. Therefore, hospital preparedness is a critical global concern that requires proactive measures to mitigate the impacts of natural or artificial disasters (5). The effects of climate change are associated with a wide range of crises, posing a great burden to health systems in many regions, especially Sub-Saharan Africa (6). The climate crisis accelerates the risk of emerging infectious diseases and extreme weather events, devastating the already weak healthcare systems. The Health Emergency and Disaster Risk Management (H-EDRM) framework emphasizes the need for disaster preparedness in all health systems. Public health systems are under severe pressure due to increasing vulnerabilities resulting from limited resources and unpredictable and rising disease burden. Most health facilities are poorly resourced and inaccessible, whereas some are severely affected by extreme weather events (7). These challenges provide an additional layer of complexities with compounding effects when the same settings must also manage a public health emergency associated with a climate crisis (8). Building a resilient health system requires an array of interconnected players to be able to safeguard the health of communities in the face of associated risks (9). Climate change is worsening the already precarious situation of health systems (6). According to the World Health Organization (WHO), the Health System Resilience Framework (7), and the new Operational Framework for Climate Resilient and Low-Carbon Health Systems, there are several key components that are vital for understanding health system resilience to climate crises (7, 9). However, there is still a paucity of scholarship addressing how healthcare facilities can be prepared for disasters.

Vectorborne diseases alone cause more than 700,000 deaths annually and contribute to more than 17% of all global infectious diseases (10). The growing concern is the change in transmission patterns of these diseases and the higher burden they represent (11). The vectors, such as mosquitoes, transmit parasites, viruses, and bacteria, posing a huge burden to the already dilapidated healthcare systems. According to WHO predictions, there will be ~250,000 annual deaths due to climate change between 2030 and 2050, an estimated 48,000 deaths due to diarrhea, and 60,000 deaths due to malaria in endemic areas (12). These diseases disproportionally affect disadvantaged people who have limited access to healthcare facilities, with dire consequences (13). Proliferation of *Anophele*s and *Aedes aegypti* mosquitoes (transmitting malaria and dengue fever, respectively) has increased due to favorable climatic variabilities, while estimates predict that global warming could lead to about a 12–27% malaria prevalence increase (14). Both transmitting mosquitoes have adapted to local human habitation, with oviposition and larval habitats in natural and artificial collections in urban and peri-urban environments (15). Approximately 390 million people are infected with dengue virus (DENV) annually, of whom 96 million develop clinical manifestations, leading to ~500,000 hospitalizations and 25,000 deaths. Viruses cause a spectrum of diseases, with symptoms ranging from mild influenza-like to severe or fatal hemorrhagic fever (16).

While the symptoms of DENV and malaria usually overlap, DENV fever symptoms last for 2–7 days. Warning signs of severe infection (feeling tired, restless, or irritable; belly pain; tenderness; repeated vomiting; epistaxis; hematemesis; or hematochezia) normally appear 24–48 h after fever (17). Laboratory confirmation is required for a correct diagnosis. However, most health facilities do not possess routine diagnostic capabilities. Other sophisticated diagnostic methods include detecting viral RNA using reverse transcription polymerase chain reaction (RT-PCR) and other nucleic acid amplification tests (17). Furthermore, there are no well-established epidemiological surveillance systems or laboratory diagnoses of DENV; hence, the inadequacy of prevalence and incidence. Malaria is routinely diagnosed by microscopy or rapid diagnostic tests (RDT). There is potential for new infectious diseases to emerge due to the geographic invasion of the pathogen, supported by climatic variability, leading to vector fecundity. This calls for preparedness, resilience, and adaptability of healthcare systems. Kenya is undoubtedly witnessing a climatic crisis. However, the extent of health facility preparedness and its effects remain poorly understood. Deficiencies in health and surveillance systems in response to these burdens are likely to contribute to the ballooning disease burden and health outcomes.

## Materials and methods

### Study site

**Figure 1:**
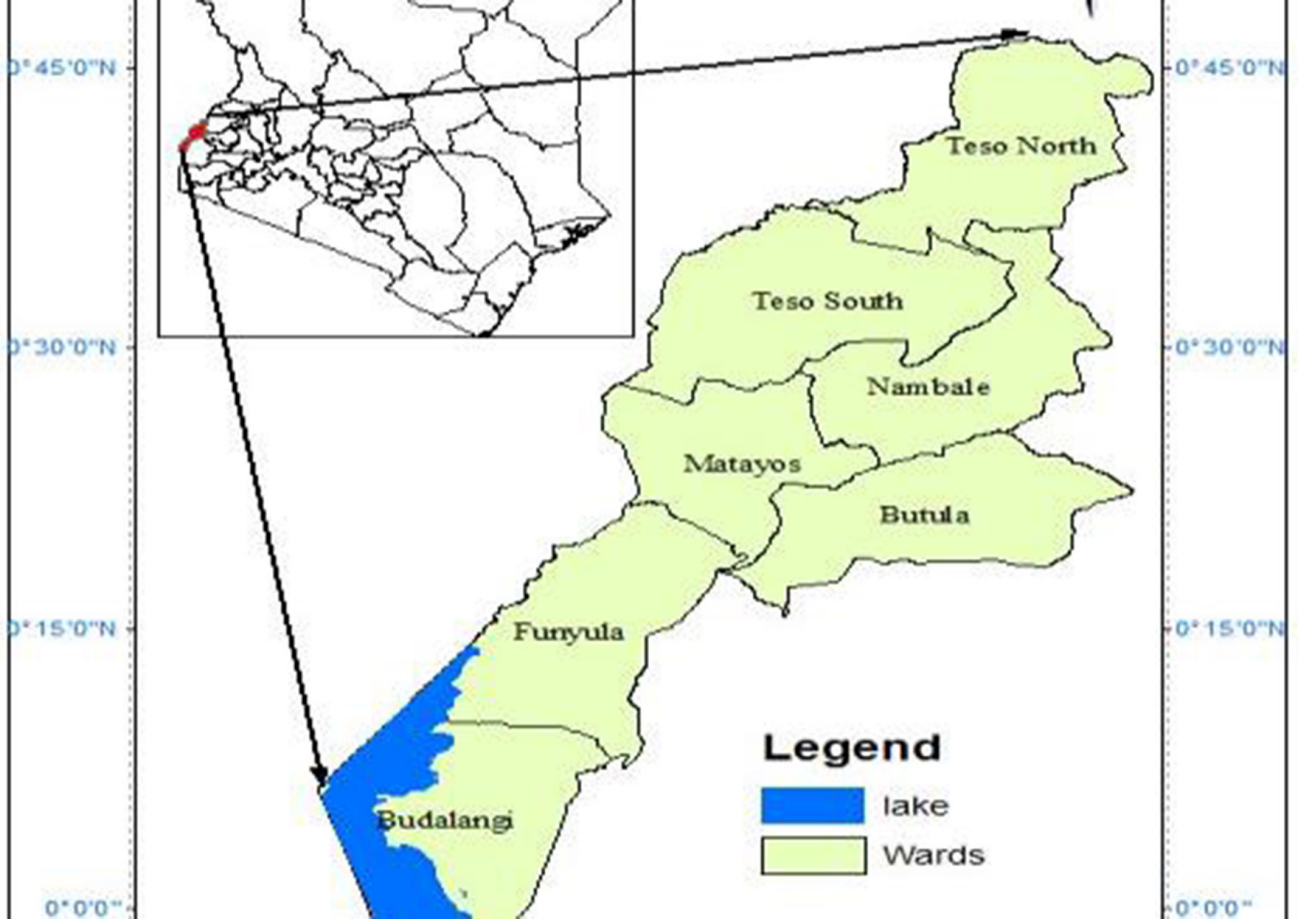
Map of Busia County.

**Figure 2:**
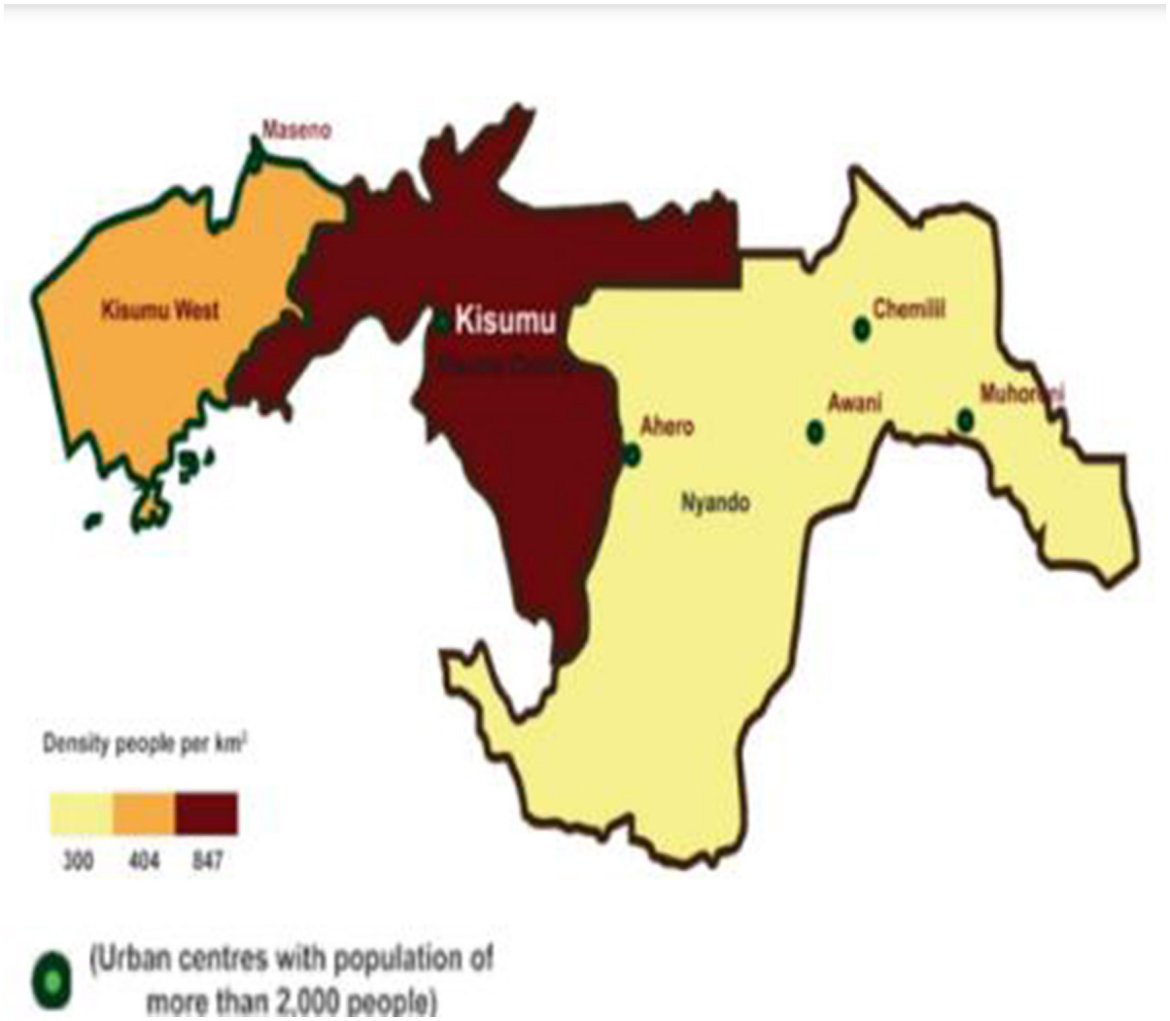
Map of Kisumu County.

This study was conducted in Bunyala Sub-County, Busia County (Figure 1), and Kisumu Central Sub-County, Kisumu County (Figure 2). The climate at both sites was modified by the presence of Lake Victoria. The counties are warm throughout the year, with temperatures ranging between 20 and 37°C. The climatic conditions at both sites are tropical in nature, and they are located in a temperate zone. Kisumu Central Sub-County is the city center and also hosts major informal settlements. The mean temperature prevailing in the city of Kisumu is 23.1°C, according to statistical data. Each year, an approximate value of 1,966 mm (77.4 inches) of precipitation. Busia County, on the other hand, is one of the smallest counties in Kenya. It is 1,198 m above sea level and is located at 0.45°N and 34.08°E. The mean temperature was 21.8°C, according to statistical data. Approximately 2,291 mm (90.2 inches) of rainfall occurs annually. Bunyala Sub-County is on the floodplain of the Nzoia River, and increased runoff from degraded catchments, coupled with climate change, increases its vulnerability to flooding disasters.

### Study design

This was a 3-month follow-up, mixed-method, cohort study among children below 5 years of age presenting with acute febrile illness (AFI) at public health facilities in Kisumu and Busia.

### Study population

The study population included children <5 years of age seeking treatment from Level 2 (dispensaries), Level 3 (health centers), and Level 4 (Sub-County hospitals) outpatient departments. Parents and the facility's health in charge were also interviewed. Key informant interviews were also conducted among key personnel under the County Health Management Committees.

### Sample size

A total sample size of 380 was calculated as per the formula shown below. Levels 2, 3, and 4 of public health facilities at the study sites were included.


$$n=\frac{\begin{array}{c} {}[Z_{\alpha }\sqrt{\left(1+1/m)\bar{p}(1-\bar{p}\right)}\\ {+Z_{\beta }\sqrt{p_{0}(1-p_{0})/m+p_{1}(1-p_{1})}]}^{2} \end{array}}{{\left(p_{0}-p_{1}\right)}^{2}}$$

where,

*n* = Calculated sample size.

*Z*_β_ = Standard normal deviate for one-tailed test based on beta level (90% power at 1.28).

*m* = Ratio of exposed to unexposed (1).

*Z*_α_/2 = Standard normal variate for level of significance (1.96).

*P*_0_ – *P*_1_ = Effect size.

*P*_0_ = The proportion of the exposed group (0.4, from previous studies).

*P*_1_ = The proportion of the unexposed group (1 – *P*_0_).

### Inclusion and exclusion criteria

Children with acute febrile illness at the OPD who tested positive for malaria or dengue virus at screening and whose parent/guardian was willing to sign an informed consent form (ICF) were included. Employees of the county government under the MoH, CHMT, CMLC, CDH, CP, or designees and meteorological officers were included in the KIIs. Exclusion criteria included any child who was unconscious or had a mental illness as determined by a clinician or Child in Care (CiC) or was involved in any malaria vaccine/drug trial study at screening time. CHMT employees who were not present during the data collection period were not interviewed.

### Sampling techniques

Both Bunyala and Kisumu Central Sub-Counties were purposively selected. Bunyala Sub-County is vulnerable to flooding, while Kisumu Central Sub-County is within the city center, hosting most of the informal settlements, and is also affected by flooding and experiencing acute water shortages during severe droughts. All L2, L3, and L4 public health facilities and their in-charge were included. Children presenting with acute febrile illness were screened for malaria and dengue fever and included in the study based on the test outcome. Members of the County Health Management Committee (CMLC, CDH, and CP) and a regional meteorological officer at the study site were purposively included.

### Data collection instruments

**Figure 3:**
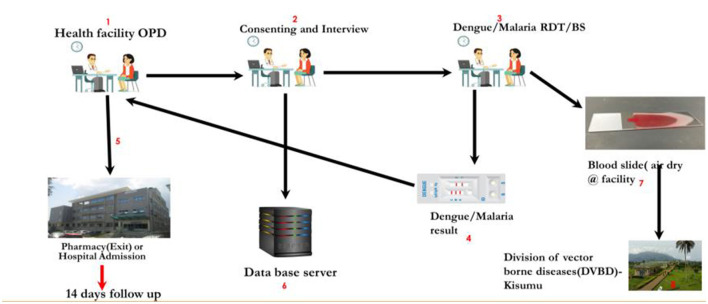
Schematic flow of events.

The study procedures were explained to the parent/guardian of the clinician at the OPD. If the parent/guardian agreed to participate in the study, they consented to it by the RA and signed the ICF. The clinician then made a study laboratory requisition for malaria and dengue virus RDT. The results were documented and made available in real time. If a child screened positive for malaria or dengue virus, then a questionnaire through the CommCare platform was administered to the parent/guardian (Figure 3). The child was then followed up with daily phone calls to the parent to collect information about the child's progress.

A questionnaire was modified from the WHO checklists for assessing healthcare facilities' vulnerabilities in climate change and was administered by the RAs to the facility in charge on their last day of data collection in the facility. KIIs were administered to selected members of the County Health Management Committee and regional meteorological representatives from both study areas.

### Data management and analysis

Data from the field were automatically transmitted via CommCare and stored on a password-protected computer. To ensure quality and completeness, there was a daily review of the database. All RAs were trained in data collection tools and diagnostic procedures to ensure validity and reliability. The tools were also validated in a different health facility before the actual data collection. Sorting and cleaning were performed using an Excel spreadsheet and then subjected to StataCorp Stata 15 (18) for analysis. Descriptive statistics were done on sociodemographic, environmental, and climatic factors. The qualitative data audio clips were transcribed, a codebook was created, and themes were created based on the codebook. Qualitative content analysis was performed using NVIVO. A code comprising one or two words describing a unit of meaning was created from the interviews after the transcription.

### Ethical considerations

All research procedures were performed in accordance with the ethical standards of the National Commission for Science in Kenya and the 1964 Helsinki Declaration and its later amendments or comparable ethical standards. This research was approved by the Institutional Scientific Ethics Review Committee (ISERC) of the Jaramogi Oginga Odinga Teaching and Referral Hospital (ref: ISEC/JOOTRH/752/23) and licensed by the National Commission for Science, Technology, and Innovation (NACOSTI, license No. NACOSTI/P/23/32018). Authorizations were also granted by the county governments of Kisumu (ref: GN133VOL.XV/250) and Busia (ref: CG/BSA/H/ADM/1/56/IX).

## Results

The average age of children was ~3.07 (1.60, 4.60) years. Of the 380 participants, 174 (46%) were female, and the majority [97 (51%)] were from Bunyala Sub-County. Out of the 206 (54%) males, 113 (59%) were residing in Kisumu Sub-County. There were more [97 (51%)] female participants in Bunyala. Most of the participants [228 (60%)] reported not having started going to school, while 152 (40%) were already attending school, 80 (42%) were residing in Busia, and 72 (38%) in Kisumu. Of those 138 (36%) who have reported having reached the primary level, 4 (44%) of them reside in Bunyala Sub-County. College or vocational training graduates were 73 (19.5%) recorded among the parents, with 49 (26%) residing in Kisumu compared to only 24 (13%) in Bunyala Sub-County at the time of data collection (Table 1).

**Table 1:** Descriptive statistics of sociodemographic characteristics across study sites.

**Variable**	**Overall (*N =* 380)**	**Busia (*N =* 190)**	**Kisumu (*n =* 190)**	***p*-value**
**Age**		3.00 (1.60, 4.00)	3.00 (1.60, 4.00)	0.7
**Gender**				**0.050**
Female	174 (46%)	97 (51%)	77 (41%)	
Male	206 (54%)	93 (49%)	113 (59%)	
**School going**				0.5
Not started school	228 (60%)	110 (58%)	118 (62%)	
Started school	152 (40%)	80 (42%)	72 (38%)	
**Highest education level**				**<0.001**
College_vocational_training	73 (19.5%)	24 (13%)	49 (26%)	
Primary and below	138 (36%)	84 (44%)	54 (28%)	
Secondary	152 (44.5%)	82 (43%)	87 (46%)	
**Source of income**				**<0.001**
**Salaried/Business**				
Yes	192(51.5%)	73 (38%)	119 (63%)	
No	188(49.5%)	117 (62%)	71 (37%)	
**Peasant farmer**				
Yes	86 (22.5%)	76 (40%)	10 (5.0%)	
No	294(77.5%)	114 (60%)	180 (95%)	
**Others**				
Yes	103 (27%)	42 (22%)	61 (32%)	
No	277 (73%)	148 (78%)	129 (68%)	

Bold values indicates statistically significant (*p* ≤ 0.05).

There is an increase in the population of mosquitoes during flooding, which transmits malaria and dengue fever associated with waterlogging. The study findings also reveal that malaria parasites thrive in areas with high altitudes and temperatures, which will lead to the spread of malaria and dengue fever.

The topography of an area, especially if it is flat and between rivers and a lake, provides many breeding grounds for mosquitoes, such as Bunyala sub-county in Busia.

“*…And then of course in the long run, of course when these floods have now been in place like that. Of course we encounter, we experience now high trends, high trends of the mosquito, of course which transmit malaria…” (KII-Busia County Malaria Coordinator)*.

“*…by just the wording of climate change. Oh… we have seen situations where the temperatures have really increased much and that at times affect the way is… it …the prevalence of the disease patterns. So at times when…We are in Kisumu by virtue that, our temperature is hot most of the time But yet we have seen cases whereby there's um the temperatures have gone down or very high and that's for sure affect how either the vector or pathogens manifest or infect people…people health…” (KII-Kisumu County Pharmacist)*.

Myths and traditional beliefs have an impact on the spread of malaria and dengue fever. Some people visit witch doctors before taking their patients to the health facilities, which is a result of a lack of awareness and public health education. Some community members still use mosquito nets to cover their gardens and have a belief that using mosquito nets causes hallucinations.

This is further supported by the fact that some malaria symptoms mimic those of the traditional signs. The disease burden should be reduced, but you find that the prevalence of malaria is still as high as 50% with RDTs and microscopy at 26.9 % cumulatively in Kisumu County. The three slums, Obunga, Nyalenda, and Manyatta, collectively stand at 25%.

“*….I think we still have issues like witchcraft issues, like probably if somebody gets sick and maybe they're so unwell, people will always want to take them to witch doctors before taking them to the facility. That is lack of awareness or lack of public health education…” “…And again, we still have issues where people continuously use their mosquito nets to cover their small gardens back at home. Some even think mosquito nets bring some hallucinations when they are asleep...” “…Some think that the mosquito nets can end up bringing bed bugs. So, we still have so many myths surrounding the issue of mosquito nets (background noise of breaking something is heard) and also other factors that are like beliefs and traditions surrounding the issue of malaria infection and the symptoms that mimic malaria...” (KII-Busia County Medical Laboratory Coordinator)*.

“*…But I think the disease burden should actually reduce, but from the data perspective when I look at my data especially from the RDTs, we are still recording a higher prevalence of almost 40% with RDTs but with microscopy we are at 26.9% but this is cumulatively. I may not actually point out and say that Obunga is contributing to this 26% per see. So cumulatively for Kisumu County, that is it. And Kisumu Central where the three slams are falling, it is not as high as compared to Seme and Nyakach and Nyando. So in terms of diseases burden, I think maybe we can rate these three slams at averagely 25%, yes, for..” (KII-Kisumu County Medical Laboratory Coordinator)*.

**Figure 4:**
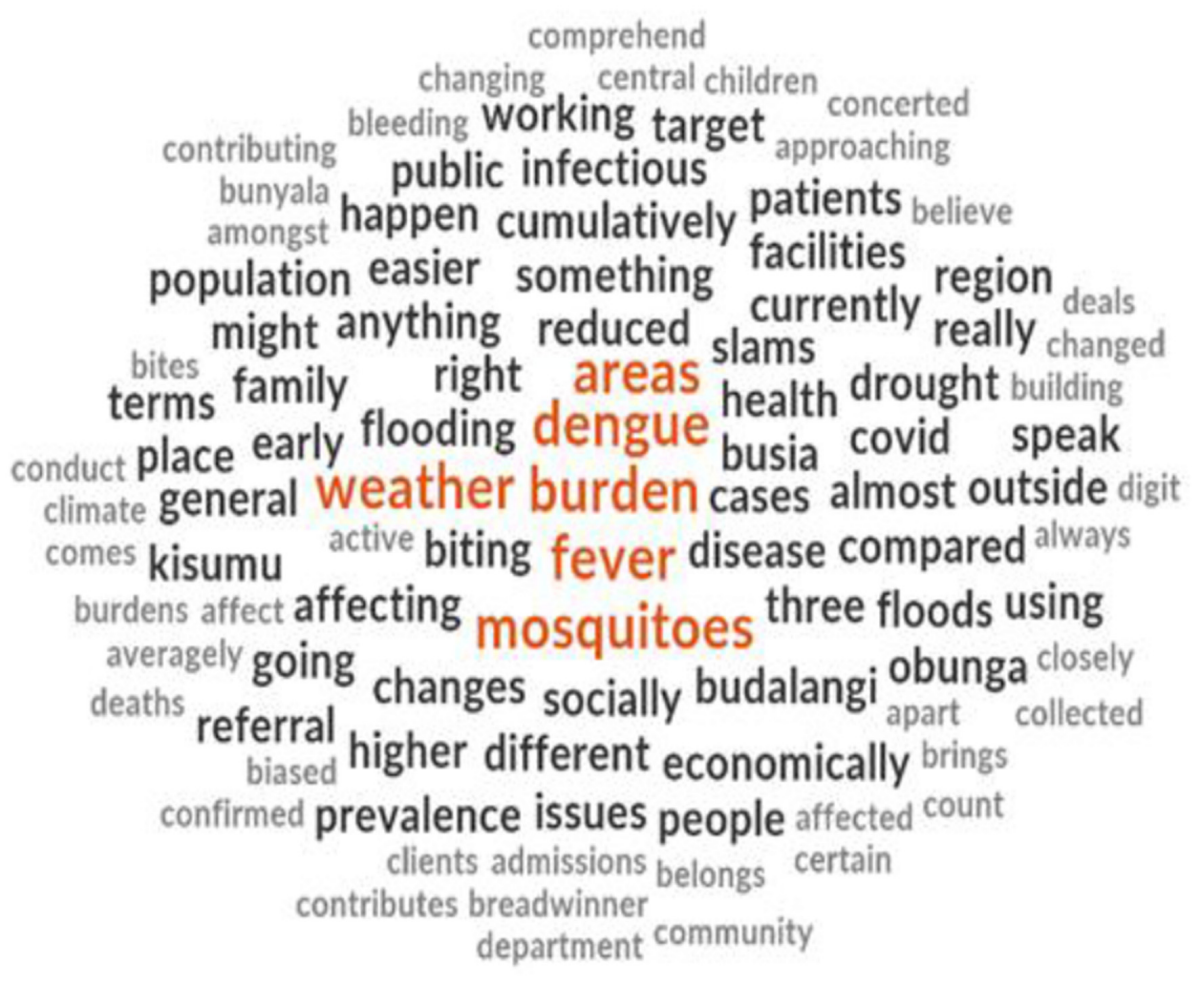
Word cloud.

From the word cloud generated from the interviews, the findings showed that the majority of the respondents talked about the burden of malaria and dengue fever. Respondents also referred to mosquitoes and weather as factors contributing to the spread of malaria and dengue fever (Figure 4).

A total of 8 (38.0%) health facilities are located on low-lying barrier islands and/or coastal regions, and 11 (52.5%) of the facilities are on or near floodplains or wetlands in both Bunyala and Kisumu (Table 2).

**Table 2:** Descriptive statistics for health facility preparedness and vulnerabilities.

**Variable**	**Overall (*N =* 21)**	**Bunyala, Busia (*n =* 10)**	**Kisumu Central (*n =* 11)**
**Is the health facility located on a low-lying barrier island**			
**and/or coastal region?**			
Yes	8 (38.0%)	4 (40.0%)	4 (36.0%)
Somewhat	1 (4.5%)	0 (0.0%)	1 (9.1%)
No	12 (57.5%)	6 (60.0%)	6 (55.0%)
**Is the facility located on or near floodplains or wetlands?**			
Yes	11 (52.5%)	6 (60.0%)	5 (45.0%)
Somewhat	1 (4.7%)	0 (0.0%)	1 (9.1%)
No	9 (42.5%)	4 (40.0%)	5 (45.0%)
**Is the facility located near major levees or dams**			
Yes	9 (43.0%)	5 (50.0%)	4 (36.0%)
No	12 (57.0%)	5 (50.0%)	7 (64.0%)
**Is the facility located near steep slopes subject to erosion?**			
Yes	4 (19.5%)	3 (30.0%)	1 (9.1%)
No	17 (80.5%)	7 (70.0%)	10 (91.0%)
**Facility is located in an area subject to fire risk**			
Yes	4 (19.5%)	3 (30.0%)	1 (9.1%)
Somewhat	3 (14.0%)	1 (10.0%)	2 (18.0%)
No	12 (57.5%)	6 (60.0%)	6 (55.0%)
Unknown	2 (9.0%)	0 (0.0%)	2 (18.0%)
**Does the facility participate in hazard mitigation plans**			
**(HMPs)?**			
Yes	9 (42.5%)	3 (30.0%)	6 (55.0%)
Somewhat	3 (14.6%)	2 (20.0%)	1 (9.1%)
No	9 (43.0%)	5 (50.0%)	4 (36.0%)
**Does the facility collect best practices and lessons learned?**			
Yes	11 (51.5%)	3 (30.0%)	8 (73.0%)
Somewhat	5 (24.0%)	3 (30.0%)	2 (18.0%)
No	4 (19.5%)	3 (30.0%)	1 (9.1%)
Unknown	1 (5.0%)	1 (10.0%)	0 (0.0%)
**Are staff adequately trained in extreme weather-related**			
**emergencies or disasters?**			
Yes	8 (37.5%)	3 (30.0%)	5 (45.0%)
Somewhat	4 (19.5%)	3 (30.0%)	1 (9.1%)
No	8 (38.0%)	4 (40.0%)	4 (36.0%)
Unknown	1 (4.5%)	0 (0.0%)	1 (9.1%)
**Are building specifications adhering to extreme climate change**			
**events?**			
Yes	9 (42.5%)	4 (40.0%)	5 (45.0%)
Somewhat	5 (24.0%)	3 (30.0%)	2 (18.0%)
No	5 (24.0%)	3 (30.0%)	2 (18.0%)
Unknown	2 (9.0%)	0 (0.0%)	2 (18.0%)
**Is water piping protected by insulation?**			
Yes	13 (61.5%)	5 (50.0%)	8 (73.0%)
Somewhat	2 (9.5%)	1 (10.0%)	1 (9.1%)
No	6 (24.0%)	4 (40.0%)	2 (18.0%)
**Does the facility campaign on water conservation awareness?**			
Yes	11 (53.0%)	7 (70.0%)	4 (36.0%)
Somewhat	2 (9.0%)	0 (0.0%)	2 (18.0%)
No	7 (33.0%)	3 (30.0%)	4 (36.0%)
Unknown	1 (5.0%)	0 (0.0%)	1 (9.1%)
**Does the facility have planning metrics for extreme**			
**weather-related events?**			
Yes	4 (19.0%)	2 (20.0%)	2 (18.0%)
Somewhat	4 (19.0%)	2 (20.0%)	2 (18.0%)
No	12 (57.5%)	6 (60.0%)	6 (55.0%)
Unknown	1 (4.5%)	0 (0.0%)	1 (9.1%)
**Is the facility a refuge for community long-term care?**			
Yes	10 (47.0%)	3 (30.0%)	7 (64.0%)
Somewhat	1 (4.5%)	0 (0.0%)	1 (9.1%)
No	10( 48.5%)	7 (70.0%)	3 (27.0%)
**Is the general facility vulnerable to EWEs?**			
Yes	12 (56.5%)	4 (40.0%)	8 (73.0%)
Somewhat	3 (14.5%)	2 (20.0%)	1 (9.1%)
No	6 (29.0%)	4 (40.0%)	2 (18.0%)
**Is there availability of a mass fatality management plan?**			
Yes	3 (13.5%)	0 (0.0%)	3 (27.0%)
Somewhat	3 (14.0%)	1 (10.0%)	2 (18.0%)
No	13 (62.5%)	7 (70.0%)	6 (55.0%)
Unknown	2 (10.0%)	2 (20.0%)	0 (0.0%)
**What is the malaria diagnostic and treatment capacity?**			
Yes	17 (81.0%)	8 (80.0%)	9 (82.0%)
Somewhat	2 (9.5%)	1 (10.0%)	1 (9.1%)
No	2 (9.5%)	1 (10.0%)	1 (9.1%)
**What is the dengue virus diagnostic and treatment capacity?**			
Yes	4 (18.0%)	0 (0.0%)	4 (36.0%)
Somewhat	1 (5.0%)	1 (10.0%)	0 (0.0%)
No	15 (72.0%)	8 (80.0%)	7 (64.0%)
Unknown	1 (5.0%)	1 (10.0%)	0 (0.0%)

**Figure 5:**
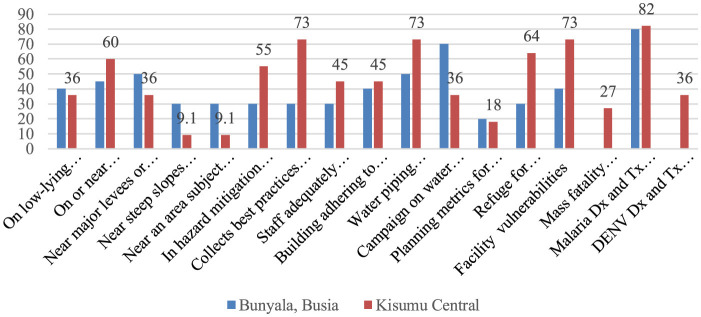
Percentage distribution of health facility preparedness and vulnerability to climate change events.

The majority of the facilities were able to diagnose and treat malaria; 81.0% of such facilities were located in Kisumu County, with only 4(18.0%) of them able to diagnose and treat dengue virus. Six (60.0%) of the facilities located on or near floodplains or wetlands were in Bunyala, while 5(45.0%) of such facilities were found in the Kisumu site. More than 8 (73.0%) facilities collected best practices and shared more lessons learned in Kisumu than in Bunyala Sub-County [3 (30.0%)] (Figure 5).

“*…You see in this country Kenya and even this county the resources are a problem. so most of the time we do climate monitoring by preparing people but when it comes then we don't have enough drugs. like recently we lacked AL and even RDTs to do the tests. We just got it some two weeks ago and we are ready to do community case management. So the resources are scars but when they come, we are ready to go out and mobilize people..” (KII-Bunyala Sub-County Public Health Officer)*.

“*…We are trying, but we are not yet there, the resources are scarce, but we are trying to ensure that we are able to*

*meet the needs of all the clients that come into the facilities. So we are not yet there, we might need more investment in terms of commodities, infrastructure expansion, health care workers with different capacities and expertise. We are not yet there, but I know something is in the pipeline and something else is happening…” (KII-Kisumu County Malaria Control Coordinator)*.

“*…On job trainings on how to handle, emerging factors especially those ones that are related to climate change. You can see now, for example there is interaction between these ministries, ministry of agriculture, ministry of energy environment and health. They normally come together even with education and discuss issues of climate change and how to mitigate them. For example the use of clean energy. Replacement of traditional cooking methods with the use of clean energy to limit those repercussions like eye infections caused by smoke. Then there is the issue of somebody dying in the house because of carbon monoxide because they are using maybe charcoal and the ventilation there is not good then somebody ends up dying. So there are always such interactions with assistants or partners…” (KII-Kisumu Central Public Health Officer)*.

From the KII extracts above, scarcity of resources negatively impacts the preparedness and response of the health department when dealing with extreme climatic conditions. Most of the time, when climate monitoring is done to prepare people, essential medical products are lacking, especially AL and RDTs. This exposes the vulnerability of health facilities by disrupting service delivery, especially in Busia and Kisumu Counties. In order to be more efficient, they suggested that more investment should be made in terms of commodity provision, infrastructure expansion, and recruiting healthcare workers who are experienced and have different capacities. Multisectoral collaborations and capacity building to ensure the mitigation of climate change challenges. For example, the interaction between the ministries of health, agriculture, energy, and the environment has positively impacted the efficiency of health facilities, especially in the reduction of infections being reported.

The other factor is that the annual work plans that the health facilities have are very key in emergency preparedness, and therefore, they have a budget for emergency drivers. This has improved the efficiency of dealing with disease outbreaks such as malaria, dengue fever, and other diseases that arise as a result of climate change. In addition, training was conducted by different sectors, where they discussed the effects of climate change and how to mitigate these effects. This enables health facilities to prepare for emergencies.

*some of the challenges faced is accessibility like movement, meaning we are not able to get all the services at the community where we move from one place to the other to seek for such services. Like when one needs microscopy, you move from one place to the other, and during flooding, such places are cut off, so such becomes a little bit difficult (KII_Bunyala Sub-County Public Health)*.

Accessibility to health facilities is also affected by extreme climatic conditions such as floods. For example, in some areas in Busia County, boats sometimes have to be used to access health facilities. Some patients are sometimes carried on their backs to get to a facility during flooding. Fatalities due to malaria infections also increase; for example, accessing a facility in the middle of the night using a boat during flooding is a problem, so a child may die in the process.

“*…Okay, our challenge will be still from my side. As I said we are getting support from UNICEF because of ah limited resources to procure those very essential commodities that's one of the challenges, so we don't have adequate resources to support fully the requirements we need for responding especially on commodities I've already mentioned diagnostics we don't have enough…”*.

Limited medical resources and commodities are also major challenges that directly affect the preparedness and readiness of medical health facilities. This is evident when the findings reveal that some facilities receive support from UNICEF to get these essential commodities.

“*…the other challenge will be staff shortage because when it comes to response to those emergencies there's a demand for human resources to respond and we have inadequacies in terms of human resources. Ah again generally from the discussion we have had the target what exactly we are targeting when it comes to climate change issues is still a challenge. Knowledge on that, generally for all of us as staff, so trainings on response to those situations what it's all about is a gap…”*.

Shortage of human resources was also a factor affecting readiness, preparedness, and response to climate change extremes because during extreme events, such as floods, health facilities become stretched, such that the available staff are not enough to handle patients. Training of staff in areas of emergency was also a mentioned gap.

“*….when we have floods, sometimes even testing these clients for malaria becomes a problem because the few MRDTs that we have are not usually adequate to test them and, in the flood, prone areas we cannot conduct malaria microscopy, so we prefer having MRDTs and MRDTs are never adequate for us during these times…” (KII-Kisumu County Medical Laboratory Coordinator)*.

Inadequate equipment in health facilities, such as microscopes, where staff have to move from hospital to hospital, becomes a challenge when there are climate extremes such as floods. Testing patients for malaria, especially during flooding, is also a challenge, because even MRDTs are not enough, especially in facilities that only perform microscopy.

Scarcity of resources in the health facilities is also a major challenge. This happens especially in cases where they receive a larger number of clients suffering from malaria in health facilities.

Lack of effective communication and connection between the meteorological department and the public health department is also a major challenge, which directly impacts preparedness and response for disease outbreaks and planning during public health emergencies. This is also evident when officials in the meteorological department have little knowledge of the impact of climate change on disease outbreaks.

**Figure 6:**
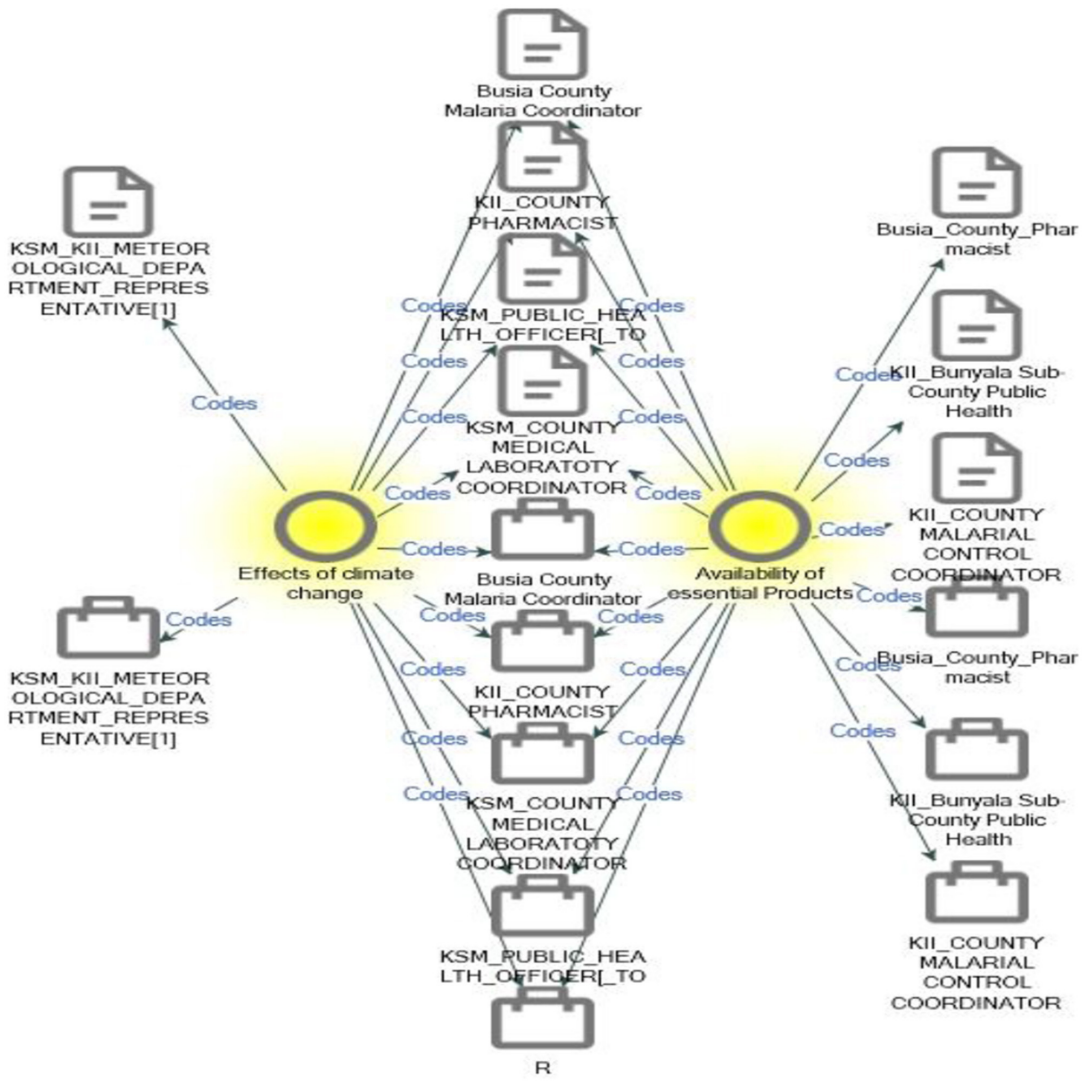
Code comparison between *c*limate change and availability of essential products.

From reference, climate change extremes and the availability of essential products confirm that climate change extremes such as floods affect the availability of essential products and services (Figure 6).

### Health outcomes

A total of 378 participants were successfully recruited and followed up. Two participants were lost to follow-up in Kisumu Central Sub-County. The overall disease recovery across both infections was 355 (93.9%), and children with *Plasmodium falciparum* infection alone had 209 (97.2%) in recovery. Readmissions were the least observed and were almost equi-distributed across the two infections. Longer hospitalization was the highest [9 (10.0%)] among children with DENV infection alone and the least [1 (1.3%)] among those with co-infections. Children with DENV were less frequently admitted compared to 3 (1.4%) among those diagnosed with *P. falciparum* alone. The proportion of readmissions was not significantly different among the infections, whereas the proportion of recovery was significantly different among the diagnoses.

A total of 184 (96.8%) recoveries were noted in Bunyala Sub-County compared to Kisumu Central [171 (91.0%)]. Longer hospitalizations [12 (6.4%)] were observed in Kisumu County. The admission of children was equally distributed [3(1.6%)] across the two sites, while readmission was slightly more at 3 (1.6%) in Busia compared to Kisumu at 2 (1.0%) (Tables 3, 4).

“*…, malaria is a killer disease but I think I might not actually what percentage per see but I know there is a small proportion of our clients that die of malaria. Uhm, in the facility, the proportion is so minimal when they come in time and most of the ones that we lose are those that are late-comers, they come late now when things are not…but in the community, when we are doing the community autopsies, you realize that people die of malaria, the children die of malaria and mother do not have, especially in the slams, the mothers do not have that, can I say the knowledge or it is just negligence, they do not, it is not their first priority to actually seek medication for malaria; and you know malaria in children mostly comes with convulsions and I think traditionally how they treat convulsions and the rest is different but by the time they will think or somebody will remind them that this is malaria, the child is already dead. So I think within the community, there are still high deaths or malaria that are being recorded and they are unnoticed and undiagnosed. But now you listen to how, what was wrong with the child, now when they are giving you the story, you just conclude as a healthcare worker that that is just malaria. But now it is not easy for me to establish what proportion, what percentage of this number that is dying because of malaria. But for those that seek medication, I think the outcome is good, over 90% that respond to treatment...” (KII_Kisumu County Pharmacist)*.

“*…Right now it depends on the time they come to the hospital. When they come to the hospital when they are not having complicated malaria, they have very quick and positive outcomes, but when they come again with strong malaria then they stay longer in the hospital and this affects them especially if they are students. They will stay away from school for some time. some women end up dying especially those who are very young and have weak immune system. so it depends on the stage of malaria but most of them fortunately they come when they have uncomplicated malaria although they cannot ignore the fact that at least one or two sometimes may come with severe irritation…” (KII_Kisumu County Malaria Control Coordinator)*.

“*…Now I'll speak of malaria It is it's good because I'll say that most of them are responding to treatments…” “….Yeah, a big percentage so, yeah a big percentage so maybe a few dies of malaria I might not give the exact percentage but generally for rural health facilities those are dispensary cell centres. We have more than 95 percent success treatment success rate because the five percent will be those who have been referred and out of the referrals maybe one to two percent deaths so can say this good response generally malaria treatment and even the deaths arise because of late referrals maybe the class came to the facility late the parasitemia is very high so those are some of the reasons why we'll have those deaths…” “…So the response is generally good and even say it's at 98% outcomes”… (KII_Busia County Pharmacist)*.

**Table 3:** Distribution of the health outcome in *Plasmodium falciparum* and dengue virus infections.

**Variable**	**Overall (*N =* 378)**	**Dengue virus infection alone (*n =* 90)**	**Co-infection (*n =* 73)**	***P. falciparum* alone (*n =* 215)**
Disability	0 (0.0%)	0 (0.0%)	0 (0.0%)	0 (0.0%)
Longer hospitalization	12 (3.2%)	9 (10.0%)	1 (1.3%)	2 (0.9%)
Readmissions	5 (1.3%)	2 (2.2%)	2 (2.6%)	1 (0.4%)
Recovery	355 (93.9%)	78 (86.7%)	68 (93.2%)	209 (97.2%)
Admissions	6 (1.6%)	1 (1.1%)	2 (2.6%)	3 (1.4%)

**Table 4:** Distribution of health outcomes by county.

**Variable**	**Overall (*N =* 378)**	**Busia County site (*n =* 190)**	**Kisumu County site (*n =* 188)**
Disability	0 (0.0%)	0 (0.0%)	0 (0.0%)
Longer hospitalization (>5 nights)	12 (3.2%)	0 (0.0%)	12 (6.4%)
Readmission	5 (1.3%)	3 (1.6%)	2 (1.0%)
Recovery	355 (93.9%)	184 (96.8%)	171 (91.0%)
Admissions	6 (1.6%)	3 (1.6%)	3 (1.6%)

The health outcomes of malaria are categorized into two types (uncomplicated and severe). There are negative outcomes, such as convulsions, if not treated promptly. The other outcomes that arise are similar to those of kidney failure. However, with the government intervention of malaria vaccines, the severity of the outcomes is in a downward trend, according to the Busia County coordinator. The other positive outcome is that children respond; more than 95% of under-five treatments are successful in rural facilities, and 2% of the treatments result in fatalities, which are mostly due to late referrals. However, if children are not able to access health facilities in time, perhaps due to flooding, it becomes a major problem because of their low immunity.

## Discussion

This study determined vulnerabilities and preparedness of public health facilities regarding the climate change impacts on VBDs. According to the WHO, the growing threat and recent epidemics of *Aedes-borne* arboviral infections in Africa have cast doubts on the adequacy of health systems to manage such disease burden (19). A study was done by Abdulwahab et al. (20). It was concluded that communities are at greater risk of acquiring diseases due to the disruption of healthcare infrastructure, exacerbation of environmental degradation, and population displacement. This finding coincides with the current study: the location of health facilities determines their vulnerability to climate change events, therefore increasing the risks of acquiring infections. Studies have also shown that flood disasters have increased in frequency and severity over recent decades, causing untold destruction to vulnerable physical infrastructure, including healthcare facilities. A study by Landeg et al. (21) determined that coastal flooding and frontline healthcare services concluded that further study is required to ensure that the healthcare system continues to review and learn from such events to increase climate resilience. The current study is in agreement with this finding: 60% of health facilities are located on or near floodplains or wetlands in the study region, exposing them to the effects of extreme weather events.

A study by Othoo et al. (22) proved that flood-risk vulnerabilities were higher in informal settlements, especially in urban informal settlements facing increased flood risk owing to their state of construction, nature of the water table, and general surface conditions. This finding agrees with that of our study, in which 40% of the health facilities were located in flood zone areas comprising urban and peri-urban settings. A proper and convenient facility location can save costs and improve the utilization of the facility. Facility location is a component of service provision assessment (SPA), an assessment of quality-of-care provision measured in health facilities across a country (23). Our analysis showed that the factors affecting the spread of *P. falciparum* and dengue fever are the availability of essential products and the effects of climate change. The healthcare sector appears to have a limited capacity to respond to weather-related impacts and is therefore unprepared for the risks associated with the ever-changing climate.

According to a study done by How (4), addressing the dangers of climate change and infectious diseases requires resilient primary healthcare systems that can respond rapidly and effectively. Several studies have also illustrated health system efforts to build resilience by mainstreaming climate change in health policies, planning, and education, indicating the urgent need to address climate-related health impacts (24). Our study also described the vulnerabilities and levels of preparedness to respond to the burden of diseases associated with climate change in Western Kenya. A review done by Lokotola et al. (25) showed that facilities and health services will be impacted by climate change and need to adapt or become more resilient to reduce the population risks and maintain service delivery despite the escalating climate change-related disruptions.

According to Braithwaite et al. (26), health systems need to be future-proofed through effective policies, adequately trained workforces, and redesigned infrastructure to meet the increasing climate change and related disease burden. The current study established that health facilities need more investment in commodities, infrastructure expansion, and healthcare workers with different capacities and expertise.

A review conducted by Biddle et al. (31) showed that ~82% of 71 empirical studies addressed resilience in the context of a specific crisis or challenge, including infectious disease outbreaks (20%), natural disasters (15%), and climate change (11%).

This review also identified a range of hazards, which included infectious diseases and general hazards. The sharp increase in diseases associated with climate change poses the risk that health systems will become dysfunctional if they are not resilient (27). Agreeing with our study, Gebremariam et al. (32) concluded that there was a high and increasing prevalence of malaria and acute dengue virus co-infection in Africa; hence, the need for resilient health facilities to handle the complications.

This study documented health outcomes in dengue, *P. falciparum, and their* co-infections, and in the malaria endemic zones of Kisumu and Busia, where AFI is the frequent reason for seeking health services for <5 years. A study by Kotepui et al. (28) concluded that physicians in endemic areas where these two diseases overlap should recognize that patients with co-infections can develop either severe malaria or severe dengue with bleeding complications. Infections caused by two or more etiological agents may modify and complicate the nature of health outcomes. The current study traced the following health outcomes: disability, readmission, mortality, and recovery. According to Engeda et al. (29), disability is a consequence of severe malaria in a significant proportion of African children. According to the current study, the health outcomes of malaria are categorized into two: uncomplicated and severe. *Other outcomes included kidney failure*. However, if diagnosed and treated well, children respond quickly to malaria. This study further revealed that some children may not be able to access health facilities in time because of flooding. According to a study by Breman et al. (30), there are up to three million deaths due to malaria and close to five billion episodes of clinical illness, possibly meriting antimalarial therapy worldwide. The current study did not record any deaths among the participants, especially from malaria, which is associated with prompt treatment availability. This was also justified by the higher recovery rate of 93.9%, as determined by the current study.

## Study limitations

This study used RDT for screening, which might not be sensitive enough to enable the detection of acute infections. Although ELISA is recommended for the confirmation of suspected dengue cases, RDTs are still useful tools for dengue screening in resource settings with limited or unavailable reference diagnostic services. Similarly, this study was hospital-based and limited to recruiting febrile children only. The findings may not be generalizable and do not reflect epidemiological status at the community level.

## Conclusion

Public health facilities are not only vulnerable but also have limited preparedness for the impacts of the climate crisis, such as infectious disease burden containment. Multisectoral and multidisciplinary policy collaboration is crucial for reducing health system vulnerabilities to withstand the complexities and burdens posed by the climate crisis. Despite the inconsistency in the supply of essential medical products, most health facilities are capable of diagnosing and treating malaria, whereas arboviral diseases, such as dengue fever, are still limited. Although this study did not record any mortality, there is an increasing burden of malaria and dengue virus co-infection in the region; hence, there is a need for improved arboviral disease surveillance. Longer hospitalization and readmissions could be associated with malaria-dengue fever misdiagnosis, leading to incorrect treatment.

## Recommendation

We recommend that public health systems align with the 2015 WHO Health System Resilience Framework in fostering health systems to prepare, respond, and recover from adverse climate events. It is also important to have a policy guide for the differential diagnosis of *P. falciparum*/dengue virus cross-infection among patients presenting with AFI. In addition, high-quality multicenter studies are required to ascertain the sero-distribution of arboviruses at the community level.

## Data Availability

The original contributions presented in the study are included in the article/supplementary material, further inquiries can be directed to the corresponding author.
